# Electroencephalographic Signal Data Augmentation Based on Improved Generative Adversarial Network

**DOI:** 10.3390/brainsci14040367

**Published:** 2024-04-09

**Authors:** Xiuli Du, Xinyue Wang, Luyao Zhu, Xiaohui Ding, Yana Lv, Shaoming Qiu, Qingli Liu

**Affiliations:** 1 School of Information Engineering, Dalian University, Dalian 116622, China; duxiuli@dlu.edu.cn (X.D.); wangxinyue@s.dlu.edu.cn (X.W.); zhuluyao@s.dlu.edu.cn (L.Z.); lvyana@dlu.edu.cn (Y.L.); qiushaoming@dlu.edu.cn (S.Q.); liuqingli@dlu.edu.cn (Q.L.); 2 Communication and Network Laboratory, Dalian University, Dalian 116622, China

**Keywords:** EEG signals, generative adversarial networks, long short-term memory network, convolutional neural networks, compressed sensing

## Abstract

EEG signals combined with deep learning play an important role in the study of human–computer interaction. However, the limited dataset makes it challenging to study EEG signals using deep learning methods. Inspired by the GAN network in image generation, this paper presents an improved generative adversarial network model L-C-WGAN-GP to generate artificial EEG data to augment training sets and improve the application of BCI in various fields. The generator consists of a long short-term memory (LSTM) network and the discriminator consists of a convolutional neural network (CNN) which uses the gradient penalty-based Wasserstein distance as the loss function in model training. The model can learn the statistical features of EEG signals and generate EEG data that approximate real samples. In addition, the performance of the compressed sensing reconstruction model can be improved by using augmented datasets. Experiments show that, compared with the existing advanced data amplification techniques, the proposed model produces EEG signals closer to the real EEG signals as measured by RMSE, FD and WTD indicators. In addition, in the compressed reconstruction of EEG signals, adding the new data reduces the loss by about 15% compared with the original data, which greatly improves the reconstruction accuracy of the EEG signals’ compressed sensing.

## 1. Introduction

An EEG signal is a random non-smooth signal reflecting the pattern of bioelectrical rhythmic activity in the brain [1], which has a significant reference value in clinical diagnosis and brain function research [2,3,4]. In the research of EEG compression sensing and emotion recognition, building a deep learning framework to train the model is necessary. However, the lack of EEG data is challenging to establish an effective, accurate and stable model. Therefore, the need for large training data remains challenging for researchers and developers [5]. Additionally, there are several restrictions on the acquisition of EEG signal data. First, the EEG signal data acquisition process requires as little noise interference as possible at the acquisition site; second, the subjects need to focus on completing a series of motion imagery or emotional performance tasks to record and classify different events [6]. Furthermore, the time during which the subjects can sustain the experiment under such a high concentration of brain energy is minimal [7]. Finally, the time cost is considerable if a standard process is followed to acquire high-quality EEG signals. Furthermore, the collected data are not always fully available because data acquisition of EEG signals is often accompanied by interference of other physiological signals such as noise and eye movement artifacts. Therefore, the data augmentation of EEG signals can expand the size of the training dataset, so that deep learning can dig deep into the internal features of the data, which will play an important role in emotion recognition, motor imagination recognition, epilepsy prediction and other fields [8,9,10]. Geometric methods like correlated combinations of initial trials, distortion and window sliding segmentation to increase the number of samples have been used by some researchers to address these issues [11,12,13,14]. Although such augmented samples can improve the classification performance, the results are unsatisfactory. In recent years, generative adversarial networks have developed rapidly [15,16,17]. They can effectively learn the distribution characteristics of real data and generate augmented samples with the same distribution. Many researchers have achieved breakthrough performance using GAN networks to augment EEG signal data [18,19].

Hartmann et al. [20] presented the first GAN framework for generating EEG signal data: the EEG–GAN, used to generate data with EEG characteristics and enhanced by the Wasserstein GAN. Abdelfattah SM et al. [21] proposed recursive GAN (RGAN), which can capture temporal features in EEG signals by replacing the fully connected layer in the GAN with the RNN. Luo et al. [22] proposed WGAN–GP to generate differential entropy features of EEG signals and used the generated differential entropy features for emotion recognition and classification tasks. A framework (E2SGAN) for synthesizing stereo electroencephalogram (SEEG) data from EEG signals was proposed in [23]. In addition, correlated spectral attention (CSA) was proposed to capture the correlation between each pair of EEG and SEEG frequencies, enhancing the discriminator of E2SGAN. The weighted patch prediction (WPP) technique was designed to ensure robust temporal results. Zhang Z et al. [24] proposed the generative adversarial network self-supervised data enhancement (GANSER) framework for data enhancement. For EEG-based emotion recognition, it combines adversarial training with self-supervised learning to produce high-quality and high-diversity simulated EEG samples. Abdelghaffar Y et al. [25] compared the performance of three different generative adversarial networks as data augmentation techniques. They investigated the effect of increasing the training data size on P300 classification performance using each GAN network. The experimental results showed that increasing the training data improves classification accuracy significantly. Zhi Zhang et al. [26] proposed the emotional subspace constrained generative adversarial network (ESC–GAN), using diversity perception loss to encourage diverse affective subspace by expanding sample differences, while boundary perception loss limits the enhancer space near the decision boundary to solve the problem of dataset imbalance, facilitating unbiased and secure EEG data augmentation.

In this paper, we propose an improved generative adversarial network model based on a combination of long short-term memory networks and convolutional neural networks to generate artificial EEG signal data more consistent with real EEG signal data. Compared with previous methods, the proposed method results in more similar distributions compared to real EEG signals. It addresses the lack of EEG signal datasets and boosts the accuracy of compressed perceptual reconstruction of EEG signals. Among them, the generator of this model consists of LSTM. The LSTM generator can extract the features of EEG data samples on time series and reconstruct the data distribution step by step to obtain the generated EEG data. The discriminator comprises a CNN rather than the forgetting unit in LSTM, which has a repeated connection structure. Therefore, the CNN can be trained faster in the case of long sequence data modeling. It can rapidly discriminate the similarity between real and generated data and direct the training of the generator based on the discriminator’s error. The public dataset of the EEG signal BCI competition serves as the basis for the simulation experiments. In addition, it is demonstrated that the proposed generative adversarial network model can learn the statistical features of the EEG signal and expand the dataset. Consequently, the EEG compressed sensing reconstruction model performs better.

The innovation and work of this study are mainly reflected in the following aspects:

- Innovative generative adversarial network model: We propose an improved generative adversarial network model, L–C–WGAN–GP, to generate artificial EEG signal data. The model uses LSTM as generator and a CNN as discriminator, combining the advantages of deep learning to learn the statistical features of EEG signals and generate synthetic EEG signal data close to the real samples.
- Data augmentation and training set augmentation: It can be used to enhance existing training sets by generating EEG data generated from an adversarial network model. This data-augmented approach can extend the scale and diversity of the training data, combined with using the gradient penalty-based Wasserstein distance as the loss function in model training to improve the performance and robustness of deep learning models.
- Applied to the compressed perceptual reconstruction model: we added the generated EEG data to the original dataset to train the compressed perceptual reconstruction model of EEG signals. Experimental results show that using the enhanced dataset can significantly improve the accuracy of compressed perceptual reconstruction, and thus improve the reconstruction quality of EEG signal data.

## 2. Related Theories

### 2.1. Generative Adversarial Network

**Figure 1:**
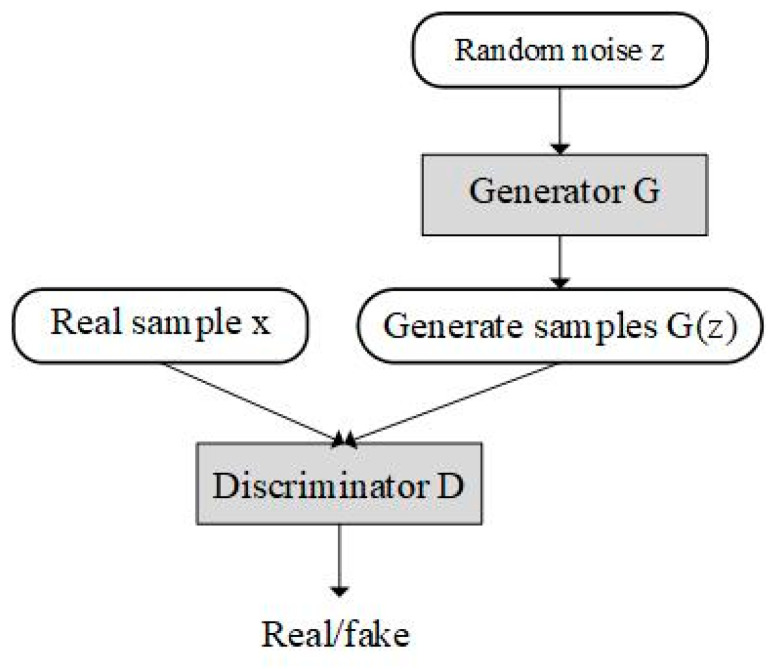
Basic GAN structure.

Goodfellow et al. [27] proposed a generative adversarial network in 2014. The GAN consists of two independent neural networks: the generator and the discriminator. The basic GAN structure is shown in Figure 1.

(1) $$\underset{G}{\min}\underset{G}{\max}V(D,G)=E_{x\sim Pdata(x)}[logD(x)]+E_{z\sim Pz(z)}[log(1-D(G(z))]\;\;\;\;\;\;\;\;$$

where *x* represents real data, *z* is random noise, *G(z)* represents generated data, *D(x)* is the probability of real data in the discriminator, *D(G(z))* represents the probability of generating data in the discriminator, *Pdata(x)* represents the real data distribution and *Pz(z)* is the generator input data distribution.

### 2.2. WGAN–GP

The GAN’s training difficulties and gradient disappearance are two of its drawbacks at times. The Wasserstein distance was introduced by an enhanced GAN known as the Wasserstein generative adversarial network (WGAN) [28,29] to address these issues. It eliminates the problem of the existence of Jensen–Shannon divergence and is utilized to measure the overlap between two distributions. The Wasserstein distance can also provide a meaningful gradient when the overlap between real and generated data distribution is small. The Wasserstein distance is defined as Equation (2):

(2) $$W(P_{data},P_{g})=\underset{\gamma \in \Pi (P_{data},P_{g})}{inf}E_{(x,y{)}^{-}\gamma }[∥x-y∥]$$

Among them, $\Pi (P_{data},P_{g})$ represents the set of all joint distributions obtained by combining $P_{data}$ and $P_{g}$, each joint distribution is represented by γ(x,y) and ∥x−y∥ denotes the distance between two samples in the joint distribution γ. The expected value of the sample distance in the γ distribution can be calculated by calculation, and the maximum lower bound of all expected values is the Wasserstein distance. In addition, the Lipschitz continuity theorem is introduced by the WGAN to resolve the issue that the Wasserstein distance cannot be directly solved. However, to satisfy Lipschitz continuity, the gradient of the discriminator is required to be no larger than a finite constant K. Weight clipping, which results in the concentration of the parameter value distribution on the two extremes of maximum and minimum. The discriminator will tend to learn simple mapping functions by this influence, resulting in poor discriminator performance.

Based on the WGAN, WGAN–GP uses gradient penalty to add constraints to the loss function, which solves the problem of gradient explosion, accelerates the training speed and makes the training more stable [30]. The WGAN–GP’s objective function is defined as Equation (3):

(3) $$\underset{G}{\min}\underset{D}{\max}L=E_{x∼P_{g}}[D(x)]-E_{x∼P_{r}}[D(x)]+\lambda E_{x∼P_{g}}[∥\nabla_{x}D(x){∥}_{p}-1{]}^{2}\;$$

### 2.3. Long Short-Term Memory Network

**Figure 2:**
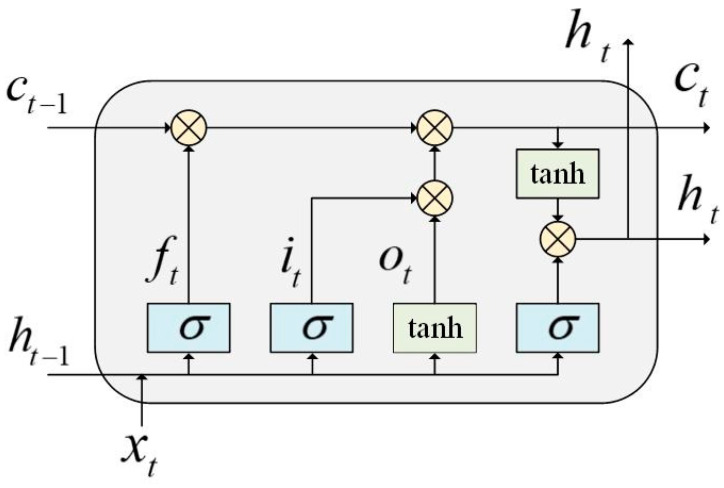
LSTM basic building blocks.

LSTM is an improvement of recurrent neural networks (RNNs) and is effective in learning sequence information at long intervals and solving the problem of gradient disappearance or explosion when training long sequence information [31]. Figure 2 depicts the structural elements of the LSTM network:

Equations (4)–(8) display the LSTM calculation:

(4) $$i_{t}=\sigma \left(W_{ix}x_{t}+W_{ih}h_{t-1}+W_{ic}c_{t-1}+b_{i}\right)$$

(5) $$f_{t}=\sigma \left(W_{fx}x_{t}+W_{fh}h_{t-1}+W_{fc}c_{t-1}+b_{f}\right)$$

(6) $$o_{t}=\sigma \left(W_{ox}x_{t}+W_{oh}h_{t-1}+W_{oc}c_{t-1}+b_{o}\right)$$

(7) $$c_{t}=f_{t}\cdot c_{t-1}+i_{t}\cdot tanh\left(W_{cx}x_{t}+W_{ch}h_{t-1}+b_{c}\right)$$

(8) $$h_{t}=o_{t}\cdot tanh(c_{t})$$

where $W_{ix},{\;W}_{ih},W_{ic},b_{i}$ is the weight parameter and bias term of input gate $i_{t}$, $W_{fx},W_{fh},W_{fc},b_{f}$ is the weight parameter and bias term of the forget gate $f_{t}$, $W_{ox},W_{oh},W_{oc},b_{o}$ is the weight parameter and bias term of the output gate $o_{t}$, $W_{cx},W_{ch},b_{c}$ is the weight parameter and bias term of the memory unit $c_{t}$ and $h_{t}$ is the LSTM network’s output value.

### 2.4. Convolutional Neural Network

**Figure 3:**
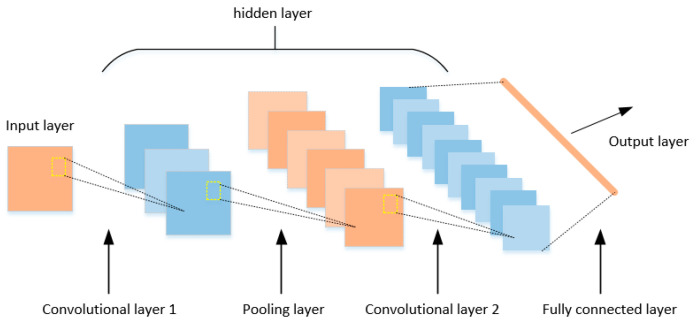
CNN basic structure.

The convolutional neural network (CNN) is a deep learning model similar to artificial neural network, as proposed by Le Cun et al. [32]. The basic CNN structure is shown in Figure 3. The CNN expands the network structure on the basis of artificial neural network, which consists of three parts: input layer, hidden layer, output layer. The input layer is used to receive the original input data. The hidden layer consists of the convolutional layer, pooling layer and fully connected layer, generating corresponding feature vectors. The internal structure of the hidden layer can modify the design until the optimal performance of the network is achieved. The output layer processes the feature vectors obtained from the hidden layer through activation functions and can be used for prediction in classification, regression or other tasks.

The core of the CNN is the convolutional layer, through which the CNN performs deep feature extraction from the input data through the convolution layer. Convolution operation is the operation of multiplying a small movable window with the input data element by element and then adding it [33]. This small window is actually a weight matrix, called a convolution kernel, and can also be seen as a specific filter. The 2D convolution can be written as Equation (9):

(9) $$(M*N)\left(m,n\right)=\sum_{i,j}M(i,j)N(m+i,n+j)$$

Adding pooling layers between adjacent convolutional layers effectively reduces the number of parameters. The main work of the pooling layer is feature selection and information filtering, as well as reducing the dimension of the deep extracted features to reduce the operation difficulty. For input maps, the output maps are generally smaller as given by Equation (10):

(10) $$X_{k}^{l}=f(\alpha_{k}^{l}down\left(x_{k}^{l-1}\right)+\beta_{k}^{l})$$

where $\alpha_{k}^{l}$ and $\beta_{k}^{l}$ are the multiplicative and additive bias terms and *down(·)* is the pooling function. The output of PL is given as an input to an FC layer.

Finally, the fully connected layer integrates the convolutional layer and pooling layer, transforming them into one-dimensional feature maps as inputs to the output layer.

## 3. Approach

### 3.1. L–C–WGAN–GP Model

**Figure 4:**
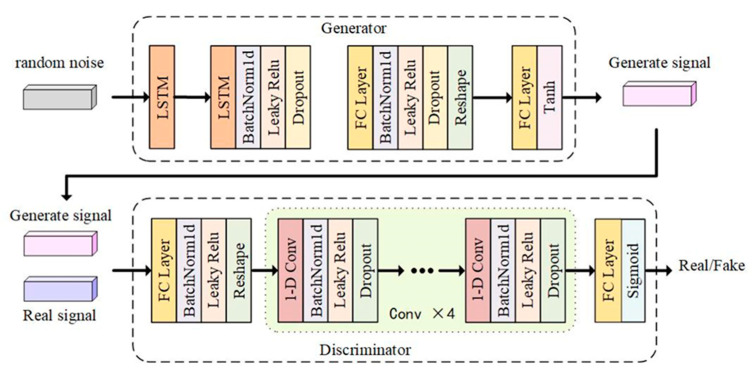
Overall structure of the L–C–WGAN–GP model.

An improved generative adversarial network model named L–C–WGAN–GP is proposed in this study. It automatically learns from existing data and generates EEG signal data similar to the original distribution. It also can retain the characteristics of real data in the generated EEG signal data. Figure 4 shows the overall structure of the L–C–WGAN–GP model. Firstly, the model inherits the idea of WGAN–GP and uses the gradient penalty-based Wasserstein distance as the loss function in model training. Secondly, the generator’s input is a noise data point that follows Gaussian distribution sampling. The generator part comprises LSTM and a full connection layer, which selectively retains historical and current information and can effectively learn the temporal characteristics of EEG signals [34]. The inputs of the discriminator are the real data and the generated data. The discriminator can be considered a binary classifier for determining whether the input sample is true or false [35]. Therefore, unlike the forgetting unit in LSTM, the discriminator component comprises a convolutional neural network without repeatable connections. The training process is usually faster when modeling long sequence data using the CNN as a discriminator.

### 3.2. Generator Design

**Figure 5:**
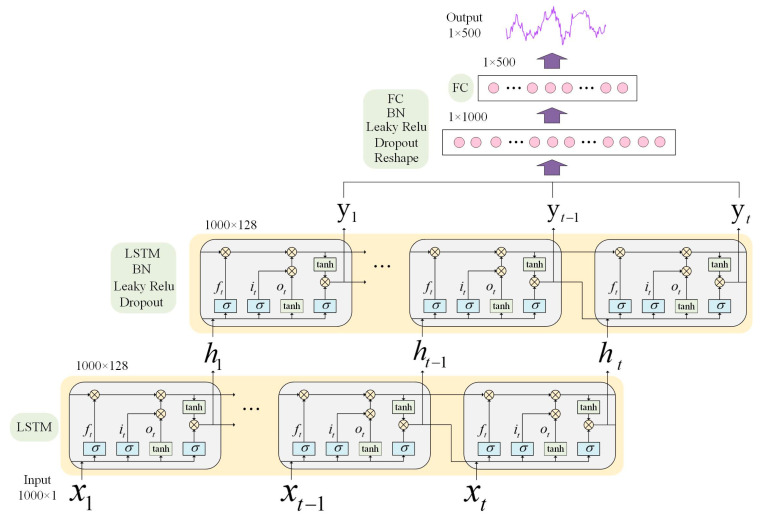
Generator structure of the L–C–WGAN–GP model.

The L–C–WGAN–GP model’s generator structure is depicted in Figure 5. The composition of the generator from bottom to top is an input layer, two LSTM layers, a fully connected layer, reshape layer, a fully connected layer and an output layer. LSTM is better able to capture long-term dependencies in sequence data, has better memory performance and can retain distant contextual information when processing signal data.

The input layer of the generator is random noise with a length of 1000 dimensions, a random sequence that obeys a uniform distribution of U [0, 1]. Each point in the 1000-dimensional input sequence corresponds to the input value of different time steps of the LSTM layer, and the time step of the LSTM layer is set to 1000. Each hidden layer cell unit of the LSTM layer receives an input data dimension of 1 × 1. The dimension of the output value ${\;h}_{t}$ of the cell unit is 1 × 128 due to the 128 neurons that make up the departmental structure of the cell unit in the hidden layer of LSTM. The LSTM layer combines the data $x_{t}$ received at t time with the output state of the cell unit at t−1 time as a new input. The output value $h_{t}$ of the first LSTM layer will be the input for the second LSTM layer at time t in this model, which sets up two LSTM layers with the same structure. After the action of two layers of the LSTM layer, a two-dimensional tensor of 1000 × 128 is obtained. Then, the output dimension of the second layer LSTM layer is reduced to a vector of 500 × 1 through the full connection layer, the Reshape layer, and the full connection layer. The Tanh activation function then processes the network output to produce the final generated signal.

Leaky Relu is the generator’s activation function, and the LSTM and full connection layers use it. The activation function’s linear slope is 0.0001. A batch normalization (BN) layer is added before the activation function to normalize the data and remove the influence of distribution offset on the input of the lower layer network [36]. After activating the function, we perform the following steps:

- Add the dropout layer to process the output of this layer;
- Set the discard rate to 0.5;
- Discard half of the network unit output in each layer;
- Set it to 0 in the discard bit, which can effectively prevent the occurrence of the over-fitting phenomenon.

### 3.3. Discriminator Design

**Figure 6:**
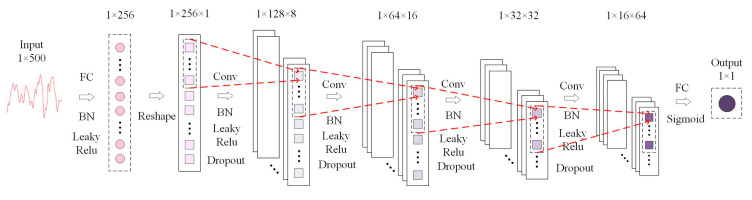
Discriminator structure of L–C–WGAN–GP model.

The L–C–WGAN–GP model’s discriminator structure is depicted in Figure 6. The discriminator comprises an input layer, full connection layer, reshape layer, four-layer convolution layer, full connection layer and output layer from left to right.

The input of the discriminator is a one-dimensional sequence with a length of 500. After a full connection layer, it is reduced to 256. Each one-dimensional sequence in a Batch undergoes a Reshape transformation to become a feature sequence with a dimension of 1 × 256 × 1 to satisfy the dimensional requirements of the convolution function for the Tensor’s input data. After the four-layer convolution layer operation, the feature sequence size is reduced by half layer by layer, and finally, the sequence of 1 × 16 × 64 dimension is output. The convolution kernel size used in the discriminator is 3 × 1, and the convolution step is 2. The convolution kernel is weighted with the input of each layer of the neural network, and the features of the output sequence of the upper layer are extracted. With the increase in the number of convolution layers, more advanced features are extracted. The four-layer convolution layer filters are 8, 16, 32 and 64, respectively. The number of characteristic graphs output from each layer increases with the number of filters layer by layer. In addition, after each convolution layer, a BN layer is added to standardize the data, a dropout layer is added to avoid network over-fitting and the discard rate is set to 0.5. The Leaky Relu function, which has a slope of 0.0001, is the activation function utilized in each convolution layer. The network’s last layer is the fully connected layer. The output of the convolution layer is mapped to a value of 1 × 1 by the fully connected layer, which is processed by the Sigmoid activation function as the output value of the discriminator. Problems involving binary classification typically use the Sigmoid function, and the discriminator’s role is to determine whether the input data are authentic. Through the Sigmoid function, the output value of the full connection layer can be mapped to a probability value in the interval [0, 1], representing the discriminant result of the discriminator for the input data.

## 4. Experimental Simulation and Analysis

### 4.1. Experimental Datasets

The BCI-IV-2a dataset contains the data of the EEG signal that records the motor imagination of the subjects. Its amplification helps to improve the recognition rate of the motor imagination, and it is recognized and widely used in the field of EEG signals. This dataset is used to facilitate the comparison of subsequent experiments. The public dataset BCI-IV-2a, which contains EEG data from nine subjects, served as the basis for producing the experimental data. In brain–computer interface experiments, subjects must perform motor imaging tasks based on arrows pointing left, right, down or up that appear on a computer screen (corresponding to the left hand, right hand, foot or tongue, respectively). In these experiments, 22 Ag/AgCl electrodes (3.5 cm distance between electrodes) were used to record EEG signals and acquire EEG signal data from 22 channels. All the signals were recorded as unipolar, with the left mastoid as a reference and the right mastoid as a grind. The signal was sampled at 250 Hz and band-pass-filtered between 0.5 Hz and 100 Hz. The amplifier’s sensitivity was 100 μV, and an additional 50 Hz notch filter suppressed line noise. The datasets can be found in the data availability statement at the end of this article.

In the experiment in this paper, the entire EEG signal is intercepted into signal frames with a length of only 2 s per period. Each of the 29,150 signal frames has a length of 500, and the quality of the generated data is evaluated using 30% of the intercepted EEG signals randomly selected as the test set and 70% as the generative model training set.

### 4.2. Experimental Environment

This paper’s computer configuration for model training is as follows: the processor is AMD Ryzen5 5600H, and the main frequency is 3.3 GHz. The graphics card is an NVIDIA Geforce RTX 3050 with 4 GB of memory. This article uses the Python 3.6 programming language and the Tensorflow 1.0 development framework to implement the model. 

### 4.3. Trial Protocol and Model Training

#### 4.3.1. Data Preprocessing

During the data pre-processing phase, the signal data were first loaded and cleaned, including replacing the NaN value with the minimum non-NaN value and adjusting the signal shape for subsequent processing. Subsequently, the signals were segmented to ensure that each segment was of a specified single length, and the processed signal fragments were integrated into a two-dimensional array. Next, the entire dataset was normalized to calculate its mean and standard deviation, and the data were normalized to ensure that the data had distributional properties of zero mean and unit variance. Finally, the dataset was divided into training and test sets based on the specified proportion of training data and shuffled using pseudorandom seeds to ensure the randomness of the dataset.

#### 4.3.2. Model Training Scheme

The training process of the generator and discriminator alternates, and the model is trained for 1000 epochs. We select a batch size of data from the real sample and mark the data from this part of the real sample as one during each training epoch; at the same time, we use the generator model to generate a batch size of the generated data, and set the label of the generated data to 0. The data of these two parts are input into the discriminant model, the loss gradient of the discriminant model is calculated and the gradient reverse update updates the network parameters of the discriminator. For the loss gradient calculation of the generative model, we set the label of the data generated by the generator to 1 and calculate the generator’s loss gradient by comparing the difference between the discriminant result of the generated data and the set label vector by the discriminator, and then fix the discriminator’s parameters and only update the generator’s parameters in reverse. Due to the significant difference in the training speed of the generator network and the discriminator network, to ensure that the two can progress together at a similar speed in each epoch, the training strategy we adopt is that the discriminator model is updated multiple times. The generator model is updated once. The discriminator is updated five times, and the generator is updated one time [37].

#### 4.3.3. Experimental Detail

The generator and discriminator selected Adam as the optimizer, the learning rate was set to 0.001 and the batch size was 64. The beta parameters of the Adam optimizer (beta1 and beta2) control the decay rate of the exponential moving average of the gradient and the exponential moving average of the square gradient. Search was performed between 0.5 and 0.9. The gradient penalty term was set to 10 and was used to control the intensity of the gradient penalty. Model training protocol uses the Step LR scheduler to reduce the learning rate to half every 50 epochs. This learning rate scheduling method helps the model to converge better during training.

### 4.4. Evaluation Indicators

#### 4.4.1. Similarity Evaluation Indicators

This paper uses three measures to judge the similarity of EEG signals to evaluate the generated EEG signals comprehensively. The three measures are root mean square error (RMSE) [38], Fréchet distance (FD) [39] and dynamic time warping (WTD) [40].

*RMSE* can effectively measure the deviation between the true and predicted values. The *RMSE* value will be lower if the generated EEG signal is more like the real EEG signal; otherwise, the *RMSE* value will be higher. *RMSE* is calculated as Equation (11):

(11) $$RMSE=\sqrt{\frac{1}{n}\sum_{i=1}^{n}{\left({\hat{y}}_{i}-y_{i}\right)}^{2}}$$

where the values ${\hat{y}}_{i}$ and $y_{i}$ are the true and predicted, respectively.

FD is used to measure the similarity between curves, which considers the location and order of the points along the curve. Smaller FD values usually represent higher quality and better diversity of the generated signals. Use $\sigma \left(P\right)$ and $\sigma \left(P\right)$ to represent the sequential set of two trajectory points, assuming that curve P has p trajectory points and curve Q has q trajectory points. Then, there are $\sigma \left(P\right)=\left(u_{1},\ldots ,u_{p}\right)$ and $\sigma \left(Q\right)=\left(v_{1},\ldots ,v_{q}\right)$. Then, you can obtain a sequence of several points: $\left\{\left(u_{a1},v_{a1}\right),\ldots ,\left(u_{am},v_{am}\right)\right\}$, and the length *‖d‖* of this sequence is calculated by Equation (12):

(12) d=d(uai,vai)i=1,...,mmin

where *d* represents the Euclidean distance, and $a_{1}=1$, $b_{1}=1$, $a_{m}=p$, $b_{m}=q$, and for any i=1,…,q, there are $a_{i+1}=a_{i}$ or $\;a_{i+1}=a_{i}+1$ and $b_{i+1}=b_{i}$. Finally, the *FD* is calculated as Equation (13):

(13) $$FD\left(P,Q\right)=min\{\left\|d\right\|\}$$

Additionally, WTD is one of the tried-and-true methods for determining the difference between two time series. A lower WTD value indicates that the two-time series differ less. In DTW, one or more time series are distorted along the time axis to achieve “alignment”, and their similarity is calculated. Suppose there are two time series, $Q=\{q_{1},q_{2},\ldots ,q_{i},\ldots ,q_{n}\}$ and $C=\{c_{1},c_{2},\ldots ,c_{j},\ldots ,c_{m}\}$, whose lengths are *n* and *m*, respectively. A matrix of n × m size can be constructed to align the Q and C implementations. Each matrix element $\left(i,j\right)$ represents the alignment of the point $q_{i}$ and the $c_{j}$, and $d\left(q_{i},c_{j}\right)$ represents the distance corresponding to the two points, calculated by the Euclidean distance. Define a cumulative distance γ, where γ(i,j) is the sum of the Euclidean distances between the points $q_{i}$ and $c_{j}$ and the cumulative distances of the smallest neighboring element capable of reaching the point:

(14) $$\gamma (i,j)=d\left(q_{i},c_{j}\right)+min\{\gamma (i-1,j-1),\gamma (i-1,j),\gamma (i,j-1)\}$$

#### 4.4.2. Evaluation Index of Compressed Sensing Reconstruction

To verify that the reconstruction effect of adding generated data to train the compressed sensing reconstruction model is superior to that of the compressed sensing reconstruction model without adding generated data, this article employs the percentage root mean squared distortion (PRD) to compare the reconstruction accuracy of EEG signals with and without generated data [41]. Equation (15) shows reconstruction accuracy:

(15) $$PRD=\frac{{∥\hat{x}-x∥}_{2}}{∥x∥}\times 100\%$$

where x and $\hat{x}$ represent the original and reconstructed signals, respectively. Reconstruction accuracy is higher when *PRD* is lower.

### 4.5. Experimental Analysis

**Figure 7:**
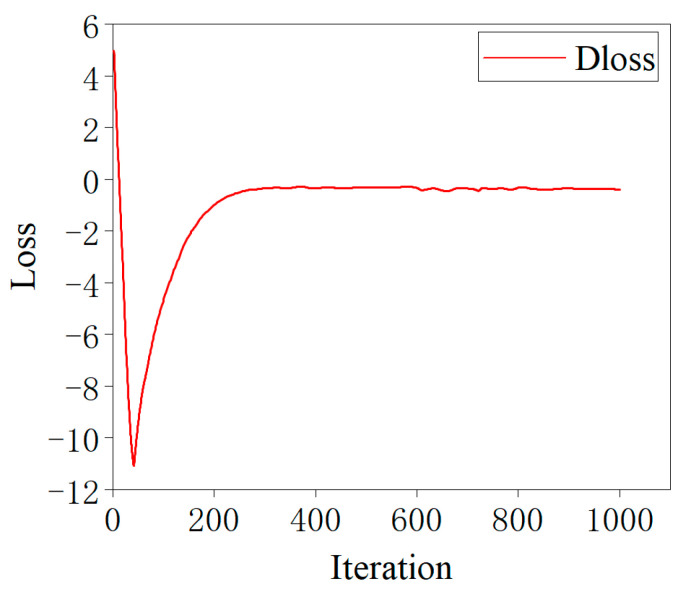
Loss curve of the L–C–WGAN–GP discriminator.

The loss curve of the L–C–WGAN–GP discriminator is depicted in Figure 7. The discriminator loss value will initially decrease significantly as the number of iterations increases, but it will soon rise above −1. After 250 iterations, Dloss rises to −0.3, indicating the network has good convergence performance. When the number of iterations reaches 600, although Dloss will oscillate slightly, it is also stable between the −0.3~0 range and continues to approach 0. The Wasserstein distance also describes the distinction between generated and real data distribution. Dloss convergence to a smaller value means that the two data distributions are increasingly similar. Furthermore, it can be observed that Dloss converges fast, and the training process is stable, thanks to the gradient penalty’s (GP) contribution to the gradient calculation process [42].

The experimental results of the deep convolutional generative adversarial network (DCGAN) [43], the Wasserstein GAN (WGAN), the Wasserstein GAN-gradient penalty (WGAN-GP) [30] and the long short-term memory generation adversarial network (LSTM-GAN) [44] are contrasted with those of the proposed method to demonstrate that the latter is more effective at producing EEG data. The generator and discriminator of LSTM–GAN mentioned above are composed of long short-term memory networks.

Table 1 shows the similarity evaluation values between the model’s real EEG data and the generated EEG data from five different models. Since DCGAN uses cross-entropy as a loss function, it is simple to produce gradient disappearance during training, which results in insufficient generator learning of the characteristics of EEG data. That is why, as seen from the table, the three similarity evaluation values of DCGAN are the largest, indicating that the model produces poor-quality EEG data. The quality of the generated data cannot be improved during training. L–C–WGAN–GP has the smallest RMSE value, FD value and DTW value compared to DCGAN, WGAN, WGAN–GP and LSTM–GAN. Therefore, the EEG data generated by L–C–WGAN–GP are more similar to the real EEG data, and the model has more advantages in learning the characteristics of EEG signals.

**Figure 8:**
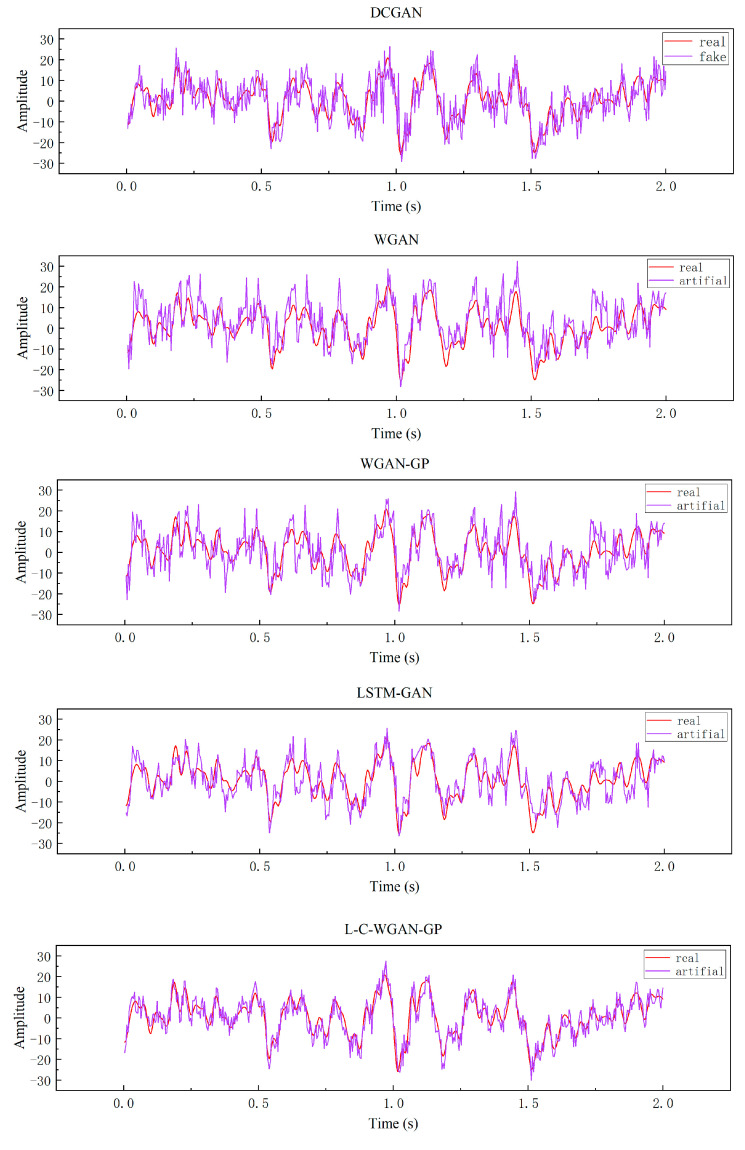
Time domain plots of generated data for five generative adversarial models.

Secondly, this paper further illustrates the above conclusions by observing the generated sample’s time domain plot and time-frequency plot. Figure 8 shows the time domain plots of the generated data for five generative adversarial models. Among them, the abscissa denotes time, the ordinate denotes amplitude and the red and purple curves denote true and generated EEG signals. As can be seen from the figure, L–C–WGAN–GP has less noise than the waveforms generated by other methods. Although the trend of all methods is basically consistent with the direction of the original EEG signal, the other methods are much higher (lower) than the original signal at the maximum and the minimum. Our proposed method is still in good agreement with the original signal at the extreme value, which is due to the fact that the LSTM network can learn temporal continuity, rather than non-continuous data, to evaluate the signal. Moreover, the EEG data generated by L–C–WGAN–GP are the most similar to the real EEG data, and their waveform characteristics are more fully learned and closer to the real waveform morphology, indicating that the model generates EEG data best. DCGAN’s EEG signal data are less effective than that of other models.

**Figure 9:**
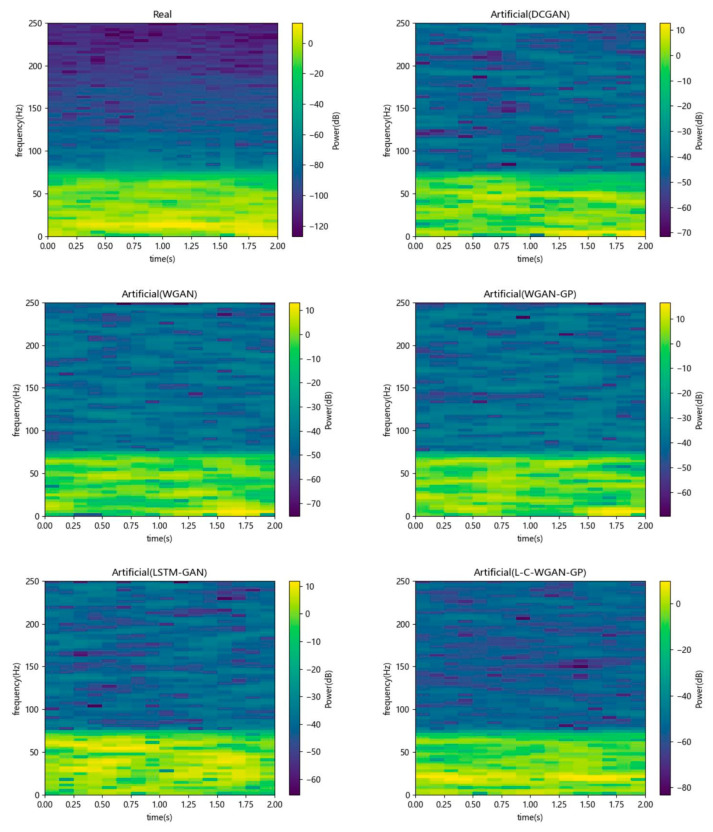
Time-frequency plots of generated data for five generative adversarial models.

Figure 9 shows the time-frequency plots of the generated data for five generative adversarial models. The abscissa represents time, the left ordinate represents frequency and the right ordinate represents power. It can be seen from the figure that the EEG data generated by the five models have similarities with the real data. Specifically, the low-frequency part has a higher power value than the high-frequency one. In addition, as can be seen in the timeline, our proposed model is also more consistent with the original EEG signal data, and compared with other methods, the power of our proposed method is as stable as the original power. Additionally, the fact that L–C–WGAN–GP’s power distribution is more comparable to the power distribution of real data indicates that the proposed model has superior performance.

**Figure 10:**
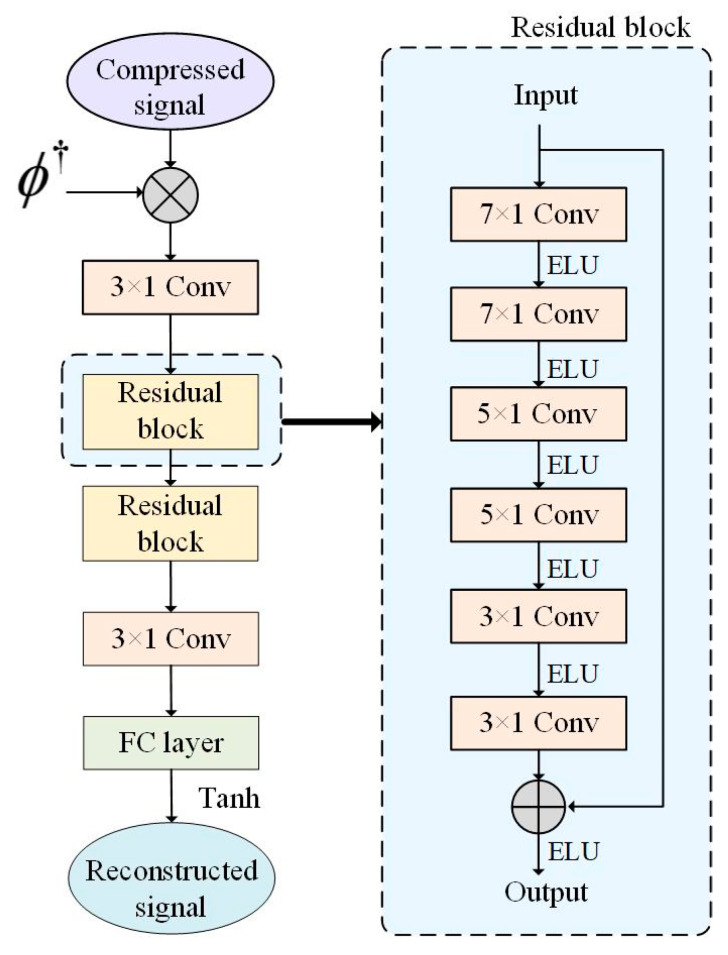
CS-ResNet compressed sensing reconstruction model.

Finally, this paper adds the generated dataset as the training set of the compressed sensing reconstruction model based on the original dataset to verify whether the added generated dataset improves the training of the reconstructed model. We utilize the network models that have been published by us as an object for the comparison of the compressed perceptual reconstruction accuracy of the EEG signals [45]. In the EEG signal compressed sensing reconstruction experiment, the length of each sample is N = 500. Utilizing the measurement matrix ϕ to project the EEG signal to a lower dimension is necessary to obtain the compressed signal with length M. The sparse binary matrix is selected as the measurement matrix, and the compression ratio (CR) is defined as $CR=\frac{M}{N}$. Therefore, this paper constructs a measurement matrix of M = 50, 100, …, 400, 450, and the corresponding compression ratios CR = 10%, 20%, …, 80%, 90%. Figure 10 depicts the compressed sensing reconstruction model for CS-ResNet.

As shown, the compressed EEG signal is first converted into 500 dimensions by multiplying it by the measurement matrix’s pseudoinverse $\phi^{†}$. The input is then converted into a 16-channel feature map using a convolutional layer with a convolution kernel size of 3 × 1 and several convolution kernels of 16. Then, through two residual blocks, each residual block contains six convolutional layers, in which the dimension size of the residual block input and output is the same, and both are feature maps of 16 channels to ensure that the input and output of the residual block in the residual learning network can be added. The number of convolution kernels in the six convolutional layers in the residual block is 32, 64, 128, 64, 32 and 16, and the corresponding convolution kernel sizes are 7 × 1, 7 × 1, 5 × 1, 5 × 1, 3 × 1 and 3 × 1. The expansion rate of each convolution kernel is set to 2. According to the size and expansion rate of the current convolution kernel, appropriate fill values are set in each convolution layer to keep the feature map’s size unchanged. The exponential linear unit (ELU) function is the activation function behind each convolution layer in the residual block. After the two residual blocks, a convolutional layer of 3 × 1 with one convolution kernel is added, and a fully connected layer is used to input the reconstructed EEG signal.

In the experiments, L–C–WGAN–GP was used to generate 20,000 generated data with a length of 500, and 25%, 50%, 75% and 100% of the total generated data were added to the original dataset, respectively, to train the compressed sensing reconstruction model fully. For reconstructing EEG signals using CNNs at various compression ratios, PRD values trained with the original dataset and PRD values trained with 25%, 50%, 75% and 100% of the generated data on top of the original dataset are all shown in Table 2.

PRD values trained with the original dataset and 25%, 50%, 75% and 100% of the generated data based on the original dataset are shown in Table 3 for reconstructing EEG signals at various compression ratios with CS-ResNet.

The table demonstrates that, when the compression ratio is 40~90%, the average reconstruction accuracy with CNN reconstruction after adding 25~100% of the generated data is 0.06~0.17% higher than that without adding the generated data. The reconstruction accuracy of CS-ResNet is 0.05~0.14% higher on average. When the compression ratio is 30%, the reconstruction accuracy after adding 25~100% of the generated data using CNN reconstruction is 0.33~1.22% higher than without adding generated data. The reconstruction accuracy of CS-ResNet is 0.3~0.80% higher. When the compression ratio is 20%, the reconstruction accuracy after adding 25~100% of the generated data using CNN reconstruction is 0.89~3.79% higher than without adding generated data. CS-ResNet has a higher reconstruction accuracy of 0.91–3.71%. When the compression ratio is 10%, the reconstruction accuracy is 1.51~5.58% higher when 25–100% of the generated data are added through CNN reconstruction than when the generated data are not added. The reconstruction accuracy of CS-ResNet is 0.87~4.69% higher. Finally, it can be concluded that the compressed sensing reconstruction model is fully trained when the number of training sets increases, significantly improving the accuracy of EEG signal reconstruction. The network model’s performance is enhanced because overfitting is avoided during training. Therefore, L–C–WGAN–GP can produce approximate EEG data samples effectively.

### 4.6. Discussion

Our proposed model can generate EEG data while maintaining the original data features, and exhibits good performance in multiple indicators, but there are also limitations. Our method uses EEG signal data as a training dataset and focuses on the enhancement of EEG motor imagination, and in the future we will try to generalize this method to more datasets for validation. The calculation quantity of LSTM in the running process of operation is significant, and GRU [46] can solve the problem of calculation quantity, but LSTM shows better performance than GRU in the experiment, and the reasons for this are worth exploring. In the future, the proposed method can also be applied to repair damaged EEG signals, after mastering the generation rules of EEG signals, to help us to further understand EEG signals and further explore the functional characteristics of the brain at the same time, in a similar field of physiological signals. For example, for nonlinear skin electrical signals, this could include ECG signal or EMG signal activity. These physiological signals have similar properties to EEG signals, are nonlinear, non-stationary and temporally related, and our method can also be popularized to augment the dataset to meet the data needs of deep learning and accelerate the development of man–machine collaboration.

## 5. Conclusions

This paper proposes a new generative adversarial network model to address the problem of EEG signal scarcity. This model can generate EEG data from the original data while keeping the characteristics of the original data. This model, based on WGAN–GP, uses LSTM as the generator and a CNN as the discriminator. The similarity index of the EEG data generated by the proposed approach is superior to that of other generative adversarial network models, as demonstrated by simulation experiments. The generated EEG signals are more similar to the real EEG signals regarding morphological characteristics. In addition, 25%, 50%, 75% and 100% of the generated data are added to the EEG signal original dataset for model reconstruction training in the EEG signal compression perception reconstruction, and the results show that the loss of compressed EEG signal reconstruction also decreases with increasing augmented data. Consequently, the reconstruction accuracy of EEG signals can be effectively improved by reducing overfitting and significantly increasing model stability. Therefore, we believe that the proposed augmentation technology of EEG signal data will have a high application value in future EEG applications.

## Figures and Tables

**Table 1:** The similarity evaluation values of RMSE, FD and WTD between EEG data and real EEG data were generated.

Model	DCGAN	WGAN	WGAN–GP	LSTM–GAN	L–C–WGAN–GP
RMSE	0.71	0.40	0.37	0.26	0.21
FD	0.99	0.92	0.89	0.81	0.75
DTW	23.71	16.89	15.23	12.87	10.38

**Table 2:** CNN reconstructs the PRD values of EEG signals at different compression ratios.

CR/%	CNN (PRD/%)				
	None	Add 25%	Add 50%	Add 75%	Add 100%
90%	0.9728	0.9291	0.8852	0.8477	0.8212
80%	1.0507	0.9913	0.9343	0.9064	0.8796
70%	1.2605	1.1954	1.1436	1.1108	1.0954
60%	1.3053	1.2589	1.1997	1.1539	1.1297
50%	1.5008	1.4321	1.3847	1.3583	1.3178
40%	2.2074	2.1282	2.0693	2.0165	1.9855
30%	6.2556	5.9215	5.7764	5.3842	5.0331
20%	25.7751	24.8785	24.0494	22.7955	21.9786
10%	44.2411	42.7291	41.2338	39.8773	38.6593

**Table 3:** CS-ResNet reconstructs the PRD value of EEG signals at different compression ratios.

CR/%	CS-ResNet (PRD/%)				
	None	Add 25%	Add 50%	Add 75%	Add 100%
90%	0.5485	0.4889	0.4465	0.4178	0.3966
80%	0.5976	0.5447	0.4979	0.4766	0.4498
70%	0.6198	0.5623	0.5244	0.4981	0.4763
60%	0.6506	0.5991	0.5493	0.5212	0.5049
50%	0.7996	0.7268	0.6881	0.6549	0.6411
40%	1.0016	0.9546	0.9173	0.8896	0.8588
30%	3.9906	3.6824	3.4564	3.3151	3.189
20%	20.1886	19.2756	18.2276	17.3934	16.4784
10%	30.2299	29.3579	27.9547	26.6872	25.5369

## Data Availability

The data used in this study came from publicly available datasets, which can be found at the following links: https://www.bbci.de/competition/iv/, accessed on 3 July 2008 (BCI CompetitionIV).
